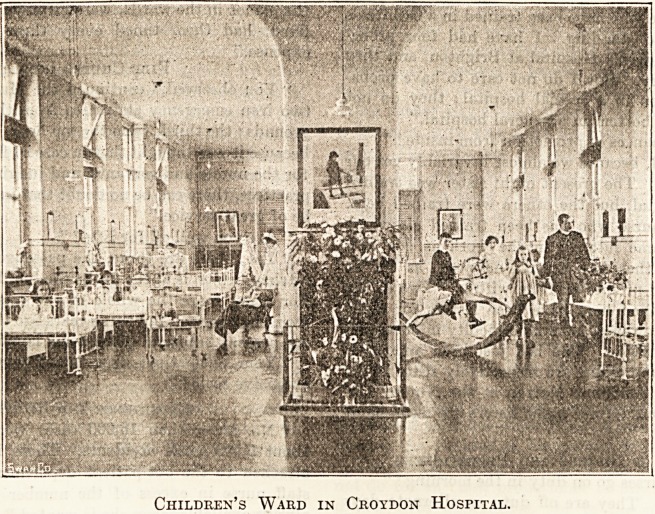# The Hospital. Nursing Section

**Published:** 1905-11-25

**Authors:** 


### The Hospital.

### Nursing Section.

Contributions for "THE HOSPITAL," should be addressed to the EDITOR, 'THE HOSPITAL", NURSING SECTION, 28 & 29 Southampton Street, Strand, London, W.C.

No. 1,000.—VOL. XXXIX. SATURDAY, NOVEMBER 25, 1905.

### Notes on News from the Nursing World.

### OUR CHRISTMAS DISTRIBUTION.

THERE is no doubt that owing to the notoriously large number of the unemployed the gifts which we are able, by the kindness of our readers, to distribute at Christmas will be even more than usually welcome to the matrons of hospitals and infirmaries. We therefore appeal to our friends to place us in a position to respond to the numerous applications which reach us for help. Christmas is rapidly approaching, and it is essential that parcels should reach us not later than Saturday, December 16. The whole of the articles presented will, as usual, be on view at the offices of THE HOSPITAL on Tuesday afternoon, the 19th, and nurses, or any contributors will be welcome any time between 3 and 5 P.M. We have to acknowledge the receipt of parcels from Miss F. A. Edwards, Ranby, Retford, and Nurse Cecil and Patients, Streatham. All contributions should be addressed to the Editor, 28 and 29 Southampton Street, Strand, London, W.C., and should have "Clothing Distribution" written outside.

### NURSES AND PARTY POLITICS.

WE observe that a meeting of the Women's Liberal Association took place at 11 Phillimore Gardens, Kensington, last week, "for the purpose of holding a discussion on the State Registration of nurses." Dr. Bezley Thorne presided. Speeches were delivered by Miss Hobbs, Secretary of the Royal British Nurses' Association, in favour of registration, and by Mr. Sydney Holland, against it. Mr. Holland, who said that thirty-five of the hospitals in London were opposed to State Registration, went on to urge that two years' training was sufficient, and observed that "at the London Hospital, the finest training home in the world, the term was two years, and they had sent a nurse to the King and she had been a satisfactory nurse." We refer to the meeting, however, at the present moment, to protest against the association of any movement in the supposed interests of nurses with party politics; and we hope that in future officials of nursing organisations will decline to take part in gatherings which are got up under political auspices, whether those of Liberal, Conservative, or any other party.

### NEW NURSES' HOMES.

WE publish to-day details of new nurses' quarters in a great London hospital and one of the larger London Poor-law infirmaries. After many days St. Mary's Hospital, Paddington, is at last in possession of adequate accommodation for the nursing staff; and the home which is attached to, but distinct from, the new Hammersmith Poor-law Infirmary at Wormwood Scrubs, will be opened next week. In both cases much care seems to have been taken to provide for the comfort and convenience of the nurses. The staff at St. Mary's Hospital enjoy the luxury of a shampooing-room, and also of heated towel-rails in the bathrooms. Each nurse has a separate bedroom, but in regard to bedrooms the Hammersmith Infirmary compares favourably with St. Mary's Hospital; for while at the latter only some of the bedrooms have a fireplace, none at the former is without this excellent means of ventilation. For the rest, there is little or nothing to choose between the two—a fact which affords one more proof of the steady improvement of nursing in Poor-law infirmaries.

### CHRISTMAS TAXES ON NURSES.

In the course of an address to the nurses at St. Marylebone Infirmary last week, Mr. Dick, Secretary of the Royal National Pension Fund for Nurses, mentioned that two sisters in two different hospitals a year or two ago withdrew their savings from the Fund for the purpose of meeting expenses in connection with the Christmas decorations of their respective wards; and "when he expostulated with one of them, her answer was that if she did not do as much as the other sisters the matron might very likely think that she was lacking in general interest in the welfare of her hospital."

### ABOLITION OF PREMIUMS AT ADDENBROOKE'S.

FOR some time past the question of abolishing nurses' premiums at Addenbrooke's Hospital, Cambridge, has been under discussion. The Committee of Management have now decided to do away with the system and to place the institution as a training-school for nurses on the same standing as other hospitals. In January 1906 the new rules and regulations will come into force and candidates for training in nursing will be received for two months' trial and if found suitable will be placed on the regular staff of nurses and paid at the rate of £5 the first year, £10 the second year, and £20 the third year, material for three uniform dresses being given. Each candidate will be required to sign a form of agreement to serve the hospital for three years. A limited number of paying probationers will be received at the rate of £1 1s. a week. A series of lectures on general, medical, and surgical nursing will be given by members of the hospital staff, and examinations will be held at the end of each course. Certificates will only be granted to nurses on the completion of three years' training. We have no doubt that the effect of the abolition of premiums, which has not been brought about without considerable difficulty, will be to increase the number of suitable candidates for admission to Addenbrooke's. Although there is a medical school attached to the hospital, the dressings and other practical work which in a large institution falls upon the students is delegated to the nurses, who thus enjoy, during training, opportunities of gaining experience that are not always available.

### THE QUESTION OF PREVIOUS TRAINING AT CROYDON HOSPITAL.

It will be seen by the report of our Commissioner's interview with the matron of Croydon General Hospital that she not only has no objection to probationers who have had previous training, but has had several, and found them quite satisfactory. It is essential that their training should have been in a children's hospital of recognised standing, and the matron confesses that she does not care about probationers who have been in a special hospital, because she finds that they do not like beginning at the bottom in a general hospital. As there are three hundred applications a year for admission, she must have an ample choice of candidates to select from. We learn that Croydon Hospital, which has nominally hitherto had ninety-five beds, though often accommodating one hundred patients, is to finally increase the total to the latter number, and it will then be fully entitled to take rank with the first-class training schools.

### THE RELIGION OF POOR-LAW NURSES.

The religious question was raised at the last meeting of the Barrow-in-Furness Guardians, at the instance of the Rev. Father Miller, who expressed a desire to move a resolution to the effect that greater facilities be afforded to the nurses to attend their respective churches on Sunday. Finding that he was not in order, he gave the needful notice and the question was adjourned. He also alluded to the test question which he said was brought up by one or two members of that Board during his absence earlier in the year. This, as far as he understood, was a suggestion that the religion of nurses should be ascertained before they were engaged. Another guardian asked if it was a legal position at all that religious convictions should be taken into consideration, and the deputy-clerk replied that "he did not think that there was any regulation." There is no doubt, however, that the regulations of the Local Government Board do not justify insistence upon the profession of a particular faith by the nurses employed.

### GROUNDLESS CHARGES AGAINST NURSES.

General statements of alleged cruelty on the part of nurses ought not to receive credence unless, or until, they have been authoritatively confirmed. At an inquest on Saturday respecting the death of a patient in the Hackney Workhouse Infirmary a witness said that the deceased woman had told her that "the nurses were most cruel to her." The Coroner having inquired if she had asked any of the other inmates about it, she replied that the nurses would not allow her to speak for fear anything should be known. A nurse from the infirmary denied that she, or any of the staff, had ever ill-treated the woman, and added that the deceased had not made any complaint; and Dr. J. W. Oliver stated that, having heard of the charges, he investigated them, "only to find that they were utterly baseless." The nurse who gave evidence thought that the mind of the deceased was wandering when she made the allegations, which is the most charitable construction that can be placed upon her conduct.

### NURSES' MISSIONARY LEAGUE.

There will be a nurses' conference in connection with the Nurses' Missionary League, in the hall adjoining the China Inland Mission House, Newington Green, from November 28-30. General meetings will take place in the evenings of the 28th and 29th at 7.45 p.m.; devotional meetings and conference in the mornings of the 29th and 30th, commencing at 10 a.m.; and in the afternoons of these days there will be social gatherings from 3 to 4.30 p.m.

### THE PRESENTATION TO MISS THOROLD.

On Thursday morning Lord Sandhurst will present Miss Thorold, the late Lady Superintendent of Middlesex Hospital, with a service of plate, subscribed to by many of the governors, nursing staff, past and present sisters, nurses, and students, as a mark of the esteem in which she is held. The interesting ceremony will take place in the Boardroom of the hospital.

### BLACKBURN DISTRICT NURSES AND THE PENSION FUND.

In the hope of exciting or stimulating interest in Blackburn in the Royal National Pension Fund for Nurses, it has been arranged for the Secretary of the Fund, Mr. Louis Dick, to give an address at 3 p.m. on Monday, December 4, in the Committee Room of the Town Hall, Blackburn. Not only nurses belonging to the District Nursing Association, but all who care to attend, will be welcomed at the meeting.

### SISTER ELIZABETH OF MIDDLESBROUGH.

On Thursday last week, the remains of Sister Elizabeth, late Mother Superior of the North Ormesby Hospital, Middlesbrough, who died on the previous Sunday, were laid to rest amid many demonstrations of affection and regret. Miss Elizabeth Canty, who was born in 1841, worked as a girl in her uncle's parish at Dover, but in 1869, hearing of the great need of helpers at North Ormesby, she offered to nurse in the Cottage Hospital, and from that time until her death she was in charge of the institution. It is related of her that old patients coming to see her, after a lapse of twenty to thirty years, invariably found not only themselves remembered, but all sorts of little personal details about their families, so much did she interest herself in those under her care. The mourners at the graveside included a great many well-known people, ministers of all denominations, and the nursing staff of the North Ormesby Hospital.

### STATE REGISTRATION.

In connection with the Nurses' Social Union in Somerset, meetings have been held at Bridgwater and Wells to consider the advantages to be derived from a system of State Registration for nurses. The chair at the Bridgwater meeting was taken by Mrs. Sanders, and at Wells by Mrs. Kennion. A number of nurses and others interested in the question attended the meetings. The speaker on both occasions was Miss Bompas, of Kensington, and her arguments were entirely in favour of State Registration.

### PROGRESS AT GLASGOW MATERNITY HOSPITAL.

In the seventieth annual report of the Glasgow Maternity Hospital, which has just been issued, it is stated that during the past year 3,456 cases were dealt with in the various departments of the hospital. In the indoor department there was an increase of ninety-nine, and nurses could not always be spared to attend to the outdoor patients who applied for assistance. The number of difficult cases, necessitating major operations, was greater than in any previous year, many of these being sent in from the surrounding districts. They included twenty-one operations of Cæsarean Section, ninety cases of contracted pelvis, and thirty-one of eclampsia. A hundred and nineteen nurses were trained in the Hospital. There is now a staff of nine midwives, seven of whom hold the certificate of the Central Midwives Board; four resident doctors, one being a lady; and an average of thirty-seven pupils. The next admission will be on January 1, 1906, and nurses are recommended in every case to take four months' training.

### DISTRICT AND PRIVATE NURSING AT COCKERMOUTH.

At the third annual meeting of the Cockermouth Nursing Home it was unanimously decided to continue to carry on the work. It was started in 1902 to provide skilled nursing for the inhabitants of Cockermouth and the neighbouring villages, and support was promised for a period of three years, which time has now expired. As it is generally conceded that the institution has amply justified its right to exist, efforts will be made to place it on a more substantial basis. Accommodation is provided for seven adults and one child. All the medical men in the town are on the hospital staff, and each attends his own cases. A Queen's nurse has been working in Cockermouth for a much longer period, her services being greatly valued by the medical men and also by the public. Since the inauguration of the Home she has boarded and lodged there, but the two funds are kept quite distinct.

### DUNDEE MATERNITY HOSPITAL.

At the recent sittings of the examiners of the Central Midwives Board, six nurses trained in this hospital presented themselves—three in London, one at Bristol, and two at Newcastle-on-Tyne—and all passed. The opportunities for practice and instruction at Dundee are excellent, and pupils are drawn from all parts of the country, who find in this vigorous school the advantage of being taught by the professors and lecturers of a school of medicine like St. Andrew's University. In addition, the arrangements and accommodation for the nurses are the same as for the nurses of the Royal Infirmary, of which the Maternity Hospital forms part.

### A HOSPITAL WEDDING.

An interesting wedding took place on Thursday afternoon last week at Sion Congregational Church, Halifax. The bride was Miss Lillian B. Williams, sister at St. Luke's Hospital, Halifax, and the bridegroom, Mr. John Harris, assistant master at the Bradford Workhouse. Miss Kidson, matron of St. Luke's Hospital, gave the bride away, and the bridesmaids were Nurse Dora Williams, of Bradford Union Infirmary, sister of the bride, and Sister Ethel Williams, of St. Luke's Hospital. They all wore their indoor uniform, and a number of nurses, also in indoor uniform, lined the aisle of the church. Mr. C. Vickers Maxwell, steward at St. Luke's Hospital, acted as best man, whilst Mr. C. E. Knowles, clerk at the same institution, presided at the organ. After the marriage ceremony, which was performed by the Rev. G. J. Williams, a light repast was partaken of at the hospital. The presents were very numerous and included a silver tea-service, half a dozen afternoon tea-spoons, and silver cake-knife from the nursing staff at St. Luke's Hospital, and a handsome oak timepiece from the staff at Bradford. Mr. and Mrs. Harris will commence their new duties as master and matron of the workhouse at Halifax on Saturday.

### QUEEN ALEXANDRA'S MILITARY NURSING SERVICE.

The following staff nurses have been confirmed in their appointments to Queen Alexandra's Imperial Military Nursing Service, their periods of provisional service having expired:—Miss S. O. Beamish, Miss A. B. Cameron, Miss K. Coxon, Miss E. M. Goard, Miss E. J. Minns, and Miss A. Rowe.

### SERIOUS IMPUTATIONS ON A NURSE.

At Tipperary Quarter Sessions a fever-hospital nurse, employed by the Guardians of Cashel Union, was plaintiff in an action for slander against the workhouse medical officer. The judge, having heard the evidence, said that the imputation that the nurse refused to do a night's duty was incorrect and slanderous, inasmuch as it might injure her chance of obtaining other employment. He awarded £5 damages, with costs. The damages are very properly small, the object in view being the vindication of the nurse's professional reputation, which is complete.

### SHORT ITEMS.

A missionary sale will be held by Miss Dashwood, Hon. Secretary of the Nurses' Union, at 5 Cambridge Gate, Regent's Park, from 3 to 7.30 p.m., on Thursday, November 30. Miss Dashwood will be "at home" to all nurses who care to attend.—The in-patients of the Cancer Hospital at Brompton were entertained on Thursday evening last week by the members of "The Butterflies." The programme included songs, monologues, recitations, dances, and quartettes.

### The Nursing Outlook.

"From magnanimity, all fear above;
From nobler recompense, above applause,
Which owes to man's short outlook all its charm."

### CHRISTMAS NOT ALWAYS A JOY.

A hospital, on Christmas Day, is often one of the brightest and happiest places a visitor can enter. There can be seen young and old giving of themselves in the days of health to minister to the happiness and the comfort of the sick. The atmosphere of such a place, on such a day, is inspiriting and cheering. Any one who has experienced the joy of such a visit will not fail to continue the practice of going to some hospital, whenever possible, at this season of the year. To hospital workers, however, Christmas time has its drawbacks, for it entails a great strain upon most of them. Unfortunately, too, as matters stand at present, it often means a serious drain upon their limited resources. This fact certainly demands the immediate attention of the chairman and members of every committee, of the honorary medical staff, and of the superintendents and matrons. It is perfectly easy, as experience has proved, for the chairman, or a few members of the committee, to raise a sufficient fund to defray the whole cost of the Christmas festivities and the decoration of the wards. Where this is not done the members of the medical staff might fill the gap, by each undertaking to defray the cost entailed so far as their own wards are concerned. In any case, it is wrong to permit the limited resources of the sisters and nurses to be trenched upon for these purposes, and any hospital where this practice prevails must be regarded as inefficient. We believe that this view will be generally held, and no doubt the abuses and hardships which exist in certain hospitals, in this connection, are due more to want of thought than want of heart. That is why we call attention to them, as well as to remind everybody concerned of the existing evils. We invite co-operation to secure that Christmas Day 1905 may be made memorable throughout our hospital system. For on that day the authorities of every well-conducted hospital may abolish every abuse of the kind for ever.

What can be said in defence of a system which was described by a correspondent to be in fact as follows? At Christmas "each sister must subscribe handsomely to the matron, who, in her turn, must give to all, each sister to her fellow sisters, and sisters and nurses alike to the whole staff, including wardmaids, porters, gate-keepers, cook, and hall-boy. In addition, each sister is responsible for the decoration of her own ward, involving plants and flowers, always costly at such a season, and she is expected to pay her share of the cost of the patients' Christmas tree, and of a tea or entertainment for the patients." To state the actual results and working of the present abuses, in this plain fashion, is to ensure their condemnation, at the hands of every intelligent person, who is responsible for the administration of a hospital in which these practices have grown up. Where they prevail, individual choice, so far as the sisters and nurses are concerned, has little or no bearing on the matter, for, as our correspondent points out, unless a nurse wishes to be boycotted altogether, she is compelled to submit to this wholesale taxation.

Dealing with the question of presents or testimonials first, we hope that every matron and superintendent will issue instructions to the whole of their staff. Let them state, it is their distinct wish, that for the future, the practice of giving presents to the officials and to individual members of the staff shall be discontinued. If they take this course they will maintain their self-respect and win the approbation of all whose opinion is worth having. Any who do less than this will in fact, show, that they are lacking in certain qualities which are essential to the proper discharge of the duties of high office, in a great institution like a hospital. It may, of course, be urged that spontaneous good feeling between officials and staff demands expression at Christmastide by the interchange of presents. Our view is—and it is based upon many years' experience—that a system which ensures the levy of money for an annual present, from one or more members of a staff to some other member, is vicious in practice. Its very monotony takes from it all spontaneity and makes it, in fact, a gross abuse, as well as a burdensome nuisance to the majority of those immediately concerned. We are most anxious to do our utmost to help to secure the immediate abolition of so bad a system. We shall be glad to hear from those institutions where the practice does not prevail, or where it may be decided to discontinue it on and from Christmas Day 1905.

There remains the question of how to provide the money for the decoration of the wards and the patients' Christmas tea and entertainments? These, in our judgment, should be provided through the agency of the chairman and committee of management, or by the honorary medical staff of each hospital. Should there, however, be any cases where special circumstances render it impracticable that either of these courses can be followed, we shall be glad to hear from the matron or the sisters and nurses of any such institution, and to receive a statement setting forth the circumstances together with details of the actual sum involved. We have confidence that our readers will co-operate, each in their own way, to secure a reform, which would make Christmas Day, in all our hospitals, a blessing and a joy to the sisters and nurses, as well as to the patients and all concerned.

### Abdominal Surgery.

By Harold Burrows, M.B., F.R.C.S., Assistant Surgeon to the Bolingbroke Hospital.

### SYMPTOMS IN ABDOMINAL DISEASE.

(*Continued from page* 86.)

### Shock.

Shock is not a symptom peculiar to abdominal disease, for it may follow severe injury to any sensitive part of the body. Yet it is such a marked feature of most acute abdominal cases that it is necessary to pay particular consideration to the subject in an article on abdominal surgery.

It has been said, aptly enough, that the nurse should develop a special sense of asepsis. Equally appropriate is it to desire a special sense to deal with the prevention of shock, for this is one of the principal duties of a nurse, and upon her skill in the performance of it many a life will depend. The truth of this should be engraved so deeply into the mind that the means of counteracting shock ought to become a habit, and at last an instinct. At the present time the instinct, and, indeed ,the habit, are rare; no doubt, because until recent years shock had received but little attention in the way of scientific research, and therefore was ill understood. But the plea of ignorance is not available now.

### What is Shock?

Shock is the condition which follows severe irritation of sensory nerves in any part of the body. It is a common experience that extensive burns, even if they are superficial, cause rapid, profound, and perhaps fatal shock. This is because a large number of the sensory nerves of the skin are injured. In the same way injury to the exquisitively sensitive nerves of the parietal peritoneum, either by acute inflammation or by manipulation during an abdominal operation, produces shock.

But there are two distinct conditions produced by severe irritation of sensory nerves and termed shock. These may be termed respectively primary shock and secondary shock. To appreciate these two conditions it is necessary to turn aside for a short while into the field of physiology. As every nurse is aware, the beating of the heart, the tension of the blood-vessels, and the movements of respiration are controlled by the vital nerve centres in the brain. Now nervous stimuli may pass from various parts of the body to these centres and so act upon them that they do not send the same impulses as usual to the respiratory and circulatory systems; with the consequence that the tension in the blood-vessels is changed, and the cardiac and respiratory movements are altered in character, or even entirely arrested.

In primary shock the effects are due to reflex inhibition of the vital centres, caused by sudden and excessive stimulation. In secondary shock, on the other hand, the vital centres have been exhausted by incessant or frequently repeated stimuli, which at any given moment have not been so sudden and so excessive as to cause reflex inhibition.

From these definitions it will be observed that primary shock and secondary shock are different conditions. The importance of the distinction in practice is very great; for, as will be pointed out presently, if a patient be suffering from primary shock, active remedies should not be attempted until he has rallied. Whereas in secondary shock the patient almost certainly will not rally but will steadily become worse, unless active remedies are applied; and the sooner operative or other drastic treatment is employed, the better will be his chances of ultimate recovery. In the one case procrastination is essential, in the other it is disastrous.

It will be observed that hitherto no mention has been made of the term collapse. This omission has been purposeful. The word is used so loosely and so variously that it causes much confusion. It is frequently applied indiscriminately to primary shock, to secondary shock, to the condition resulting from copious hæmorrhage, and to other bodily states; and those authors who have tried to confine the term to a single condition, differ in their opinions as to which one it ought to be applied.

### Primary Shock.

A plunge into cold water or a sudden fright are familiar examples of this, in a mild form; the gasp and the bounding heart-beat which follow are the effects of the sensory stimulus in the one case and the mental stimulus in the other, upon the respiratory and vaso-motor centres in the brain.

A stronger stimulus causes more striking effects, and if sufficiently severe or sudden the respiration and heart-beat may stop. In other words, complete reflex inhibition of the vital centres may occur. Sudden death from shock caused in this manner, is not very uncommon.

If the injury be less sudden and severe, a milder and incomplete form of inhibition is produced. In this the vital functions are not arrested entirely, although they are reduced in intensity. The heart still beats, but with little vigour, and the pulse at the wrist is soft and, perhaps, imperceptible; the respirations are shallow, the surface of the body is cold and clammy. This is the common form of primary shock which is so frequently present in patients when they are admitted to hospital after a severe accident. The condition is usually present as an immediate consequence of severe abdominal injury, and occasionally in acute abdominal disease, for example, strangulated hernia.

The patient may die without recovering from this state of primary shock. More often his general condition gradually improves; and he may forthwith get well if the injury has been a trifling one, such as a blow on the abdomen without laceration of any of the viscera; or he may gradually pass into the condition of secondary shock. Usually there is a distinct interval between the early and the late shock; although sometimes the former state may pass imperceptibly into the latter.

It is an interesting fact that if primary shock be sufficiently pronounced—and this will depend largely upon the suddenness of the injury and the intensity of the mental impression by which it is accompanied—the patient feels no pain. At the most he is but dimly conscious of his surroundings; and although he may be moaning as if in great agony, yet he is not in pain.

This freedom from pain in the presence of severe and perhaps fatal injury is remarkable. The phenomenon may be observed, not only in the presence of shock, but also in certain cases of abdominal injury where there is no primary shock. For example, rupture of an abdominal organ may not give rise to either pain or shock until some hours have passed by. Thus, a boy who had been knocked down and run over by a brougham, walked to his home a quarter of a mile away and declared he was quite unhurt, and yet he died within thirty-six hours of a complete rupture of the intestine. In another instance a child with a lacerated spleen was able to sit out and enjoy a school-treat tea, and to eat several large pieces of cake, before any symptoms of serious internal injury arose. The explanation of such cases is that the sensitive parietal peritoneum has escaped injury, and wounds, however extensive, of the insensitive viscera do not give rise to pain or shock until peritonitis has set in.

It may be added that patients differ widely in their susceptibility to shock; and the degree of shock induced is by no means a measure of the amount or gravity of the damage sustained.

### Treatment of Primary Shock.

Only first-aid treatment is required during the period of primary shock. The less interference there is beyond what is absolutely necessary, the better. If there be a wound an antiseptic dressing must be applied, without elaborate endeavours at cleansing the wound or the skin in its neighbourhood, and hæmorrhage must be arrested by pressure or other suitable means. The chief thing is to make the patient warm and keep him at rest until he revives. He should not be washed or disturbed in any way until the primary shock begins to pass off. Operations in this stage are commonly fatal, and are seldom attempted. Stimulants may be ordered, but the nurse should not administer them on her own initiative, as they may do more harm than good.

### Summary.

Primary shock is due to reflex inhibition of the vital centres in the brain, caused by sudden stimulation of sensory nerves. (1) The inhibition may be complete, so that sudden death is brought about. (2) The inhibition may be incomplete and momentary, as in the case of a plunge into cold water. (3) The inhibition may be incomplete and more lasting, as occurs in most cases of severe injury. First-aid treatment is all that should be attempted so long as the primary shock lasts; and the patient should be kept warm and absolutely undisturbed until he has rallied.

### The Nurses' Clinic.

### POINTS IN THE NURSING OF CHILDREN.

Perhaps one of the most disagreeable duties of a nurse is the cleaning of heads infested with pediculi. But since it is a duty which every nurse has to fulfil at some time or other, it is as well to consider the best method of dealing with this very common difficulty. Of course, this state of things is found chiefly among the poorer classes, who naturally have not much time and attention to bestow upon the toilet of their children, but it is not always such a culpable sign of neglect as it looks. Children of a weakly constitution are much more inclined to this affliction than strong, hardy ones, and as long as children sit close together on school benches, so long will it be impossible to prevent the spread of this disorder. Pediculi find their way into the heads of even high-school children, and from common experience we know that servant girls of the rougher sort are very often victims to this scourge through their own neglect and uncleanliness. Old people, too, who have no one to look after them, and whose senses have become somewhat dulled, often suffer from this unpleasantness. I have known an old woman of seventy-six who really required to have her head shaved to eradicate the persistent dwellers in her hair. To avoid the very beginnings of such an experience it is a wise precaution to daily comb the heads of children who are attending school, and of children who are not strong enough to go to school. Strange as it may seem, I know that even in a hospital ward it is very possible for these creatures to spread from bed to bed in a most unaccountable way. Whenever possible it is wise to keep separate combs and brushes, and to wash children's heads once a week. Pediculi have a knack of multiplying in a most appalling manner, and as every nurse knows who has had to deal with these cases, the small nits which are really cemented to the hair are more difficult to get rid of than the pediculi themselves. First, then, as to nursing treatment. Let the head at bedtime be thoroughly combed, and, if the hair is long and the child is young and the attack is a bad one, cut the hair as short as at the nape of the neck. Then thoroughly soak the hair in carbolic oil and cover the head with a white cotton cap, a handkerchief tied at the four corners in schoolboy fashion will answer the purpose admirably, and having spread some old cloth over the pillow, put the child to bed and leave further operations till the morning. The next day wash the head thoroughly with a solution of borax to get rid of the oil, and henceforth systematically wash the head once or twice a day with warm vinegar and water. The vinegar softens the cement which fastens the nits to the hair, and at every washing a quantity of nits will be seen on the top of the water. If the case is very obstinate some time will have to be spent daily in detaching the nits with the fingers, and in vigorous brushing of the hair; but perseverance will be rewarded, and in a week or two it is possible to see excellent results. It is a well-known fact that the irritation produced by "pediculi capitis" results in a tiresome form of eczema, which covers the scalp and affects the ears as well. When such is the case the head must be treated with linseed poultices for the softening of the scabs, and when these are removed a non-irritant ointment must be applied to heal the sores. These cases are very tedious, but they can be cured, and a little quiet talk with the mothers of the patients is sometimes indicated. It is a useful precaution to keep the nails of all children short, as scratching the scalp is apt to produce sores, and germs flourish under the finger-nails. In a general way soft soap is the best kind for washing children's heads; the strong pungent odour of it is a wholesome deterrent to pediculi, and it does not stick to the hair like

**THE NURSES' CLINIC**—*Continued.*

ordinary soap. When in a hospital ward nurses should put some kind of gloves upon the patient's hands to hinder all possibility of scratching; a piece of lint roughly shaped to the hand and tied on to the wrist with a piece of tape can be made use of in an emergency. "Prevention is better than cure" is a wise old saw, and certainly much trouble would be saved if those who have the care of children took pains to prevent the invasion of pediculi.

### Incidents in a Nurse's Life.

### THE DOCTOR'S MISTAKE.

"Good morning, nurse, are you at a case? I am in want of someone to take charge of an old lady—only for a few days—double pneumonia. Soon be all over, I am afraid; left it too late. Awful house, too, will you come? . . . Afraid she's pretty poor, though, only called me in at last moment, can you help me? You are just the one I want."

I was greeted thus, all in a breath, when taking a walk one frosty morning, by a doctor whom I knew very well, and had often worked with. Of course I said I would go, and about an hour later was knocking at the door, armed with my bag, containing several things I expected I should find wanting from what Dr. —— had said, and so thought it wise to bring with me.

I was shown in by a very dirty charwoman, who called noisily at the foot of the stairs to someone above, and in response a child of thirteen or so came out, and meeting me took me into the sick-room. Never shall I forget it; a bed, and on it the "old lady," and all else in the room a perfect jumble. It had been left two days previously by the workmen, half-way through whitewashing and papering. In the middle of the work the "old lady" had suddenly collapsed, and becoming speechless they had laid her on the bed and sent for the doctor, but had attempted nothing more.

Setting to work with the aid of the child, who I found had sat up the two nights, "as the doctor said someone was to," and the charwoman only came in the day-time, I began a hunt for towels and a few things to use to begin operations at once. But everything was packed away owing to the workmen, and I discovered the girl knew nothing, being only a neighbour's child lent for a day or two when the catastrophe happened. So I descended to the kitchen and burrowed there. After packing my patient up in turpentine stupes as directed, putting her into a little order, I worked away quietly but steadily till a bright fire, steaming kettle, and screens of clothes-horses and newspapers, made my queer room quite presentable.

I soon learnt the little "old lady" lived there alone, save for two elderly gentlemen boarders, and a tiny terrier who shared her all with her. The only servant was the daily charwoman.

She was very well preserved. Her hair was black and thick, her eyes keen, and the bright colour, the outcome of a temperature of 104°, made her look quite juvenile and pretty.

In the passage I encountered one of the elderly boarders, very much alarmed and intent upon beating a hasty retreat from this house of distress. But it dawned on me that this might mean disaster in a financial way should my patient possibly recover, so I talked to him calmly and reasonably, and assured him that I would do my best to make him comfortable "until" my patient was better, and so he consented to bide a wee, and I felt that one trouble at least was tided over.

How we struggled through that desperate fight, the doctor and I, without a soul to help, to come near, to take any interest in her life or death, I can now hardly realise. The boarders' rooms I tidied, and ordered in such things as were needed for their table, for I had found a purse in my patient's bedroom with a very small amount in it. Likewise a tiny screw of paper, with an address on it, which was to play an important part after. I wrote to the address, thinking it might be a relation or a friend, but no answer came.

. . . . . . . . .

It was about two weeks after my coming, and my patient had delighted the doctor and myself by actually pulling through, and was now quite out of danger. I had before told her who I was; how my home was near, and I had been taking care of her, and that the boarders and her dog were well cared for; and I now asked her, seeing she was better, a few questions, laughingly telling her that she was a perfect marvel with such eyesight and teeth at her time of life.

"Why, nurse?" she said. "Well," I replied, "I tried to take your teeth out when you were so ill, and found they were your own, and you know how surprised the doctor was at your not needing glasses, it is indeed wonderful at your age!"

Noting that she did not reply, and fancying perhaps that she was tired, I left her to rest a bit, and gave myself up to dreaming by the fire as to who she could be. Unmistakably a lady; but so terribly poor. The post brought an answer at last, or helped me to arrive at one, for a letter came requesting the nurse to send the Christian name of the patient she had written about to the same address as was on the scrap of paper, stating the age and any other particulars which could be gleaned. I lost no time in doing so, though I learnt afterwards how easy it is to be deceived in guessing a sick woman's age.

I told my patient a little of what I was doing, and then it all came out. Such a sad story, a runaway marriage, a scoundrel husband, seclusion from friends, ending with a piteous cry: "Oh! they must never know where I am, nor how placed, it would shame me to death!"

. . . . . . . . .

But they did. They drove up one day, and I had the consolation of seeing my "old lady" dressed in a trim coat and skirt, and a smart little toque, going off with them once more to visit at her old home.

She was only thirty-seven after all. Illness had deceived my good medical man, and to this day he smiles when we mention Mrs. ——, whose lungs have quite healed, and who is never tired herself of teazing the doctor about the very old lady who called him so suddenly in, and who had never had an illness in her life until then.

### To Nurses.

We invite contributions from any of our readers, and shall be glad to pay for "Notes on News from the Nursing World," "Incidents in a Nurse's Life," or for articles describing nursing experiences at home or abroad dealing with any nursing question from an original point of view, according to length. The minimum payment is 5s. Contributions on topical subjects are specially welcome. Notices of appointments, letters, entertainments, presentations, and deaths are not paid for, but we are always glad to receive them. All rejected manuscripts are returned in due course, and all payments for manuscripts used are made as early as possible after the beginning of each quarter.

### The Nurses of Croydon General Hospital.

INTERVIEW WITH THE MATRON. BY OUR COMMISSIONER.

On the occasion of my visit the other day to Croydon General Hospital I was struck by the fact that, though situated almost in the centre of a great town, it possesses a beautiful garden, adorned by several fine trees, and that not only the wards, but all the rooms in the building, enjoy the maximum of light. The architect appears to have had the exceeding good sense to have introduced windows in every possible place, with the result that there are no dark corners in the hospital, notwithstanding that the first portion of it was erected nearly forty years ago, the extensions having been carried out at different dates.

Going round with the Matron, Miss Mary Bird, I commented on the number of chrysanthemums and plants which adorned the wards, and she rejoined that they were always well supplied by their own gardener, who takes the greatest interest in his work, and helps to make the place bright at all seasons of the year. When we had been through the wards, which are on two floors, and the nurses' quarters, which are on the top floor of the hospital, and, though not a separate home, are not lacking in either convenience or comfort, I asked the Matron how many beds there are supposed to be in the hospital.

"We formally return the number 95," she replied, "but, as you can judge for yourself, each ward containing 20 beds will easily hold 25; and, as a matter of fact, with couches, which we frequently use, we generally make up 100 beds. There are actually four large wards containing 20 beds, and four smaller ones with 15, but it is, I believe, intended immediately to make the addition of five permanent beds."

"Has the hospital been enlarged since you came here?"

"Yes. When I came here 11 years ago there were 84 beds. The building was originally a private house, to which two wings were added, the first in 1882 and the second in 1894. Subsequently, in 1900, the main block, which was the private house referred to, was pulled down, and the present administrative block, with a southern entrance, was erected in its stead. We have, as you see, plenty of space for further extension, and as the population of Croydon is still rapidly increasing, and we seldom have a vacant bed, it will no doubt soon be necessary to make another enlargement."

Children's Ward in Croydon Hospital.

### The Training.

"Did you introduce the three years' training?"

"No; it was introduced four years before I came, but the work has doubled since I came here. The great advance of surgery has enormously increased it. I knew something of Croydon before my appointment, because I was Matron of the Nurses' Institute in the town for 16 months."

"Where were you trained?"

"At Worcester and Leicester General Infirmaries. I then worked for the Worcester Nursing Institute for several years, and as a nurse I preferred district to hospital work. District nurses, too, I think, do more good than any others; but hospital life is certainly more cheering, and I cannot say that I have any cause to regret the fact that I was chosen for my present post. In some respects it is an advantage to be the Matron of a comparatively small hospital. It is true that as I have no assistant matron or home sister, I find plenty to do, but on the other hand I have the satisfaction of knowing all the members of the staff individually and being able to exercise constant personal supervision. The nurses may come to consult me whenever they like during the week, and the only time when I exclude them from my room is between 2 and 3 on Sunday afternoon.

### Three Hundred Applications a Year.

"How many nurses are there?"

"Six sisters and 16 probationers. I have more than 300 applications each year, as against five vacancies, for probationers. This gives me a very wide choice, and enables me without difficulty to select educated girls. Even then there are eligible candidates always waiting for admission."

"I suppose they come for a month on trial?"

"Yes, but in our next rules the time will be increased to two months. I should like it to be three months, because I am convinced that it takes that period for the Matron to judge

THE NURSES OF CROYDON GENERAL HOSPITAL—*continued.*

the capacity of a girl, and the candidate to judge whether the work is too hard for her. But a two months' trial is absolutely necessary. This hospital is not large enough for preliminary training, or else I believe in the value of it."

"Is there any special feature about the training?"

"The practical portion in the wards is, of course, given by the sisters, and a series of lectures on physiology and anatomy by the medical members of the staff; there are also nursing lectures. Every second year cookery classes are held, at which all the probationers attend, the sisters during that time being on duty alone. At the end of the three years, after examination, the certificate is given."

"What are the age conditions?"

"Candidates should be not under 22 or over 27, and I prefer them to be 24. I think that the age might be extended to 30. The salary the first year is £10, which covers the premium of £10 which each probationer pays, £14 the second year, and £18 the third year. Both indoor and outdoor uniform are provided."

### The Question of Previous Training.

"Do you object to candidates who have had previous training?"

"Not at all to those who have been trained in a children's hospital of recognised standing. I have had four probationers from the Children's Hospital at Brighton, and they were quite satisfactory. But I do not care to have probationers who have been in a special hospital; they do not like beginning at the bottom in a general hospital."

"Are there good chances of promotion from inside?"

"Very good indeed, because we almost invariably get our sisters from the staff. The present night sister was trained at the London Hospital, but the others were trained here. In my opinion the nurse who has been trained elsewhere does not take the same interest in the work as our own nurses, and I have therefore always adopted, as far as possible, the policy of promotion from within. Two of the nurses who were trained here have been sisters for eight years. Our nurses, however, have no difficulty in obtaining good appointments outside. One has just been appointed sister at Colchester, and others are respectively sister at the Poplar Hospital and Assistant Matron at the Nurses' Co-operation. Two of our former night sisters are matrons of cottage hospitals."

### The Relation of Sisters and Probationers.

"When do your nurses go on duty in the morning?"

"At eight o'clock. They are off duty on alternate days from 2 to 5; on the other days they leave the ward at 7 instead of 8.45 Every alternate month they have 24 hours off duty; the probationers have three weeks' holiday in the year and the sisters a month."

"What about the sisters' hours of duty?"

"They are the same as those of the probationers. When I came the probationers had to be on duty at 7 in the morning, and I altered the hour to 8, because I think that as the probationers have to work under the sisters the latter should be on duty at the same time. I have a strong feeling as to the importance of the entire staff working in harmony, and I have breakfast with them four mornings in the week at 7.30, and preside at the first or second dinner every day. One of the sisters always presides at the supper, and tea is the only meal the probationers take alone."

### The Appreciation of Responsibility.

"Are your meals at night at all troublesome?"

"In the absence of care a difficulty may easily arise, but there is no need why it should. Given a little variety there is no dissatisfaction. The night sister is responsible for these meals."

"How many nurses are on duty during the night?"

"The night sister, a probationer for each large ward, and one for the three small wards. During the day a sister, a staff nurse, by which I mean a probationer in her third year, and a probationer are on duty in each ward. The sisters do a lot of practical work here, and I leave them entire control of the wards. My experience is that this responsibility tends to make them more conscientious in the performance of their duties."

### The Nurses' Quarters.

"With regard to your nurses' quarters, I notice that every bedroom has a fireplace."

"That is a matter to which I attach great importance. It is essential to ventilation, and if a nurse be slightly indisposed it is a great comfort for her to be able to have a fire in her bedroom. Every nurse has a room to herself, and also several of the maids."

"The nurses' sitting-room appears to be exceedingly comfortable."

"Yes, and likewise the dining-room, which is a recent acquisition, the old dining-room being given to the domestic staff. The piano you saw in the nurses' sitting-room, and all the pianos in the wards, were gifts, and the donor, while he lived, had them tuned every three months at his own expense."

### Fire Chutes and Lifts.

"You observed," continued the Matron, "that we have two iron emergency staircases from the first floor into the grounds; the third, from the top floor, has lately been added because it was thought that in case of fire danger might arise for the nurses and servants, and judging from experimental practices, there can be no doubt that in case of need it would be of great service."

"Are you well off for lifts?"

"Yes; there are three, the passenger lift, the lift to the kitchen, which is at the top of the building, and the coal lift. For many years the whole of the washing was done away from the hospital, but three years ago the Committee gave us our present beautiful little steam laundry."

"Your out-patient department appears to be on a very extensive scale?"

### Experience and Recreation.

"No fewer than 15,000 cases were treated last year; about 5,000 small accidents. There is no nurse specially attached to the out-patient department, but we have one staff nurse in excess of the number required, and she is always available when she is wanted."

"Is any dispensing done by the sisters?"

"No; I do not see how it could be arranged, but where it is feasible I think it is an excellent plan. As it is we are always very busy here. Even the garden, much as we value it, adds to the work; on fine days as many as 12 beds are moved out. Last year we had upwards of a thousand in-patients, and although only a country hospital, the opportunities offered to the nurses of gaining experience during their term of training do not by any means compare unfavourably with those of larger institutions. They have also the advantage of recreation out of doors, such as tennis and croquet, while indoors the manager of the Grand Theatre sends me every Monday some tickets for their use."

### Where to Go.

November 24, 25.—American Bazaar, Town Hall, Stratford, E., for West Ham and East London Hospital Extension Fund. December 1.—London Homœopathic Hospital, Great Ormond Street, W.C. Sale of fancy articles and pictures left over from the garden fête at Kensington Palace Gardens.

### St. Mary's Hospital, Paddington.

### THE NEW NURSES' HOME.

After a long period of building operations the Home for the nurses of St. Mary's Hospital, Paddington, is an accomplished fact, and it occupies the top floor of the new Clarence Wing. Most of the rooms overlook Praed Street, but a few face the old wing of the hospital. This is the quietest portion of the new home, but owing to the elevated position the constant traffic of the streets below is scarcely noticeable.

The top floor is reserved entirely for night nurses, the second floor appropriated to the probationers, while the first floor is almost exclusively for sisters and staff nurses. The Home is connected with the hospital proper by a covered bridge with seven windows on each side. At the entrance of the Home there is a small room set apart for nurses, where they can see their friends in their off-duty time. Altogether there is accommodation for about one hundred and sixteen nurses. The home sister, the out-patient sister, and the office sister have cosy little sitting-rooms next to their bedrooms; the other sisters have sitting-rooms off their own wards. The corridors and some of the bedrooms are heated by means of radiators; there are small fireplaces in the other bedrooms. The whole of the building is lighted by electricity. There is also an electric lift from the basement to the top floor, but up to the present it has not been used. On each floor there are five bathrooms, very comfortably arranged, with spacious baths and including heated towel rails. On the second floor there is a shampooing room, provided with a rose and a row of basins so as to facilitate the necessary process of hair-washing.

Each bedroom is fitted with a spring bedstead, a chest of drawers with looking glass attached, a marble-top washstand with a cupboard of two shelves, a large wardrobe with a deep drawer underneath, and a chair. The sisters are allowed, in addition, a small writing table. All the bedroom floors are covered with linoleum. Each floor is provided with a gas ring and kettle, where all the nurses have the privilege of making tea during certain hours of the day, provided that each clears up as she goes, so that no traces of tea-making may be found in bedrooms or elsewhere. In order that there may be no difficulty about this a pail is even provided as a receptacle for the tea leaves, so that there may be no excuse for putting them down the sinks.

On the second floor there is a large class-room, where all lectures and examinations are held, and where nurses may study in quietness, their studies being assisted by the use of a library of educational books. The general library for the nursing staff consists of a judicious selection of the standard authors, fiction, biographies, and travels, as well as various magazines and papers. The books are kept clean in handsome glass bookcases. On each floor there are large box-rooms and store-rooms to enable a nurse at any time to get to her box without trouble. The probationers' sitting-room is also on this second floor, a large room with plenty of light and air, a fireplace at each end, several easy chairs, and a piano. The staff nurses' sitting-room is on the first floor; it faces Praed Street, and has a small balcony, which will be much used in fine weather by nurses in their off-duty time. The room has five windows, draped with peacock blue curtains, there are two large fireplaces, three couches, and a comfortable Chesterfield sofa, all upholstered in a pretty shade of blue. There are writing tables and octagonal tables, rocking chairs, easy chairs, chairs of all descriptions, a piano, and an additional home-touch is given by the pictures on the walls and the vases well filled with flowers on the mantelpieces. Leading off the third floor is a flat roof, from which, on a clear day, there is an extensive view, including St. Paul's and the Crystal Palace.

The appointments of the Home seem to have afforded great pleasure to the inmates. There is only one drawback to their satisfaction, and it is that the new wards beneath them are still empty for want of funds, and appear at present likely to remain so.

### Hammersmith Infirmary.

### THE NURSES' HOME.

The new Hammersmith Union Infirmary, which Princess Henry of Battenberg will open on December 1, is situated in Wormwood Scrubs, and faces a wide expanse of fields and trees with the town roofs and chimneys in the background. It stands cheek by jowl with the prison—a pleasing contrast. Both on Monday were wrapped in sunshine under a cloudless azure sky, when even the latter could not look gloomy, while the Infirmary, with its red brick walls, white stone facings and spacious windows, seemed hospitable and imposing.

The Nurses' Home is built just apart from the Infirmary, and is reached by several covered paths. It is intended to accommodate eight sisters and 40 nurses, the workhouse attendants having their quarters in the workhouse. The bedrooms are uniform, those of the sisters being rather larger than those of the nurses. They are all a fair size, with nice large windows and pretty fireplaces in each. The walls are distempered in a soft shade of terra-cotta, and when all the arrangements are completed the rooms will be fitted with electric light.

There are three bathrooms on each floor and a spacious linen-room well-fitted up with shelves. On the ground floor there are three sitting-rooms for the use respectively of the sisters, charge-nurses, and probationers; the prevailing colour is green and terra-cotta, the windows are large and light, looking out over the Scrubs, and the rooms will doubtless be very attractive when furnished. Opposite the sitting-rooms there is a fair sized lecture-room for the nurses.

The corridors are wide, have polished block floors, and are heated with hot-water pipes, a coloured glass inner door making the corridor on the ground floor very effective.

The nurses' dining-room is in the Infirmary, and it will not do for any new probationer to flirt with time when the dinner hour approaches, for to the uninitiated the path which leads to it is quite bewildering. The room itself is large and pleasant, having five windows on one side, a fireplace at either end, two entrance doors, and a convenient small kitchen and pantry combined at the far end.

At present the Home is not occupied by the nurses, nor is it furnished, and many details are incomplete, but when it is quite ready for them they will doubtless find it both charming and comfortable.

### Midwives at St. Paul's Cathedral.

Shortly before three o'clock on Tuesday afternoon some sixty midwives and nurses assembled on the west front steps of St. Paul's Cathedral in response to the kind invitation of Miss Gregory for a look around the crypt before the meeting in the Chapter House at 3.30. In the room, which was crowded, they were received by Miss Gregory, Dr. Annie McCall, and others. Dr. McCall, who presided, opened the meeting with a collect, and then gave an address. She spoke earnestly on the necessity of example, especially of women, in the matter of temperance, and though not a pledged abstainer herself, she had been always impressed with the number of cases, medical as well as surgical, that were distinctly traceable to drink. For twenty years she had been working in South London, and she claimed to know something of the ways of the London poor. She believed that at the time of their confinements the mothers were peculiarly impressed with what the midwives said.

Miss Gregory followed with an animated speech, in which she deprecated the manner in which midwives are treated. Referring to her own experiences in Somersetshire she said that when she went to one confinement, a well-meaning individual brought a bottle of gin and one of whisky to her. "Now, nurse, which will you have, and which for the patient?" When she was told that the midwife was an abstainer, and the patient for the time being would be likewise, she replied cheerfully: "So much the more for me," and proceeded to take alternate tastes neat from each bottle in turn!

Dr. Mary Rocke spoke of the faith that sick people have in those on whom they are dependent, and stated that one of her hospital patients had been told that if she had remained in her home for her illness her mother had promised her a bottle of brandy, which would ease her of all pain. This, Dr. Rocke said, was partially true, as if in a state of intoxication at the time, she might not suffer pain. But she would feel worse afterwards, and possibly be quite ill.

Subsequently many midwives joined the Total Abstinence League, and seemed proud of the pretty badges of the league, and then the cloaked and veiled visitors trooped down the old oak stairs to partake of afternoon tea dispensed by willing assistants.

### Appointments.

[No charge is made for announcements under this head, and we are always glad to receive and publish appointments. The information, to insure accuracy, should be sent from the nurses themselves, and we cannot undertake to correct official announcements which may happen to be inaccurate. It is essential that in all cases the school of training should be given.]

Chulmleigh Cottage Hospital.—Miss Lucy C. Baily has been appointed nurse-matron. She was trained at the Royal Devon and Exeter Hospital, Exeter, and has since been staff nurse on holiday duty at the East London Children's Hospital, Shadwell; staff nurse at the Royal Hospital for Diseases of the Chest, City Road, London; sister of the men's and children's wards at the Victoria Hospital, Folkestone; and charge nurse at the Brixham Cottage Hospital and District Nursing Institution, where she has taken matron's duties.

Epsom Workhouse Infirmary.—Miss Edith Kate Bennett has been appointed charge nurse. She was trained at Nottingham Workhouse Infirmary, where she has since been staff nurse. She has also been nurse at Windsor and Kingston Poor Law Infirmaries.

Infirmary, Mayday Road, Thornton Heath.—Miss Emily Northover has been appointed assistant matron. She was trained at Middlesex Hospital, and has since been sister at Bethnal Green Union Infirmary, and night superintendent at Croydon Union Infirmary.

Winwick County Asylum, Warrington.—Miss L. A. Grice has been appointed second matron. She was trained at Guy's Hospital, London, and has since done private and district nursing. She has been sister at Graylingwell Hospital (West Sussex County Asylum); assistant matron at St. Edmundsbury Private Asylum, Dublin; and assistant matron at Salop and Montgomery Counties Asylum, Shrewsbury.

Wolstanton and Burslem Union.—Miss Marion Beaumont has been appointed superintendent nurse. She was trained at Crumpsall Infirmary, Manchester, and has since been charge nurse at Park Hospital, Hither Green.

### Everybody's Opinion.

[Correspondence on all subjects is invited, but we cannot in any way be responsible for the opinions expressed by our correspondents. No communication can be entertained if the name and address of the correspondent are not given as a guarantee of good faith, but not necessarily for publication. All correspondents should write on one side of the paper only.]

### UNTRAINED NURSES AT BRIGHTON WORKHOUSE INFIRMARY.

The Secretary of the Workhouse Infirmary Nursing Association writes: In reference to your Note on the appointment of untrained workhouse nurses, it may be well to point out that the latest Order on nursing—1879—only requires that the nurse should have "practical experience" of nursing; no mention is made of training, except in the case of a superintendent nurse, who alone is required to have three years' qualification. Compared with this standard, the Irish Nursing Order—1901—shows a distinct advance when it demands that the training be in an efficient school for medical and surgical nurses.

### A NURSE'S EXPERIENCE IN YORKSHIRE.

"A Lover of Justice" writes: Seeing in The Hospital a trained nurse's experience in Yorkshire, I am writing to say that I have been in both fever and convalescent general work, and although never having had any complaint as regards my work, and obtaining a thoroughly satisfactory reference from my doctor, for some unexplained reason or other the matrons did not honour me with the same, notwithstanding that I resigned of my own accord. Therefore I think that if matrons studied the welfare of their nurses, both trained and untrained, more than they do, it would be better for the nursing world in general.

### A WEAK SPOT IN GIRLS' EDUCATION.

"M. E. S." writes: As a constant reader of your columns and in reply to "E. M. M.," who writes in last week's issue "A Weak Spot in Girls' Education," I should like to say that good practical experience can be had in the care and management of babies and young children in sickness and health at one of the nurseries, where infants are received a few weeks old, in varied conditions. Girls and young ladies are received as paying probationers for one year more or less, according to circumstances. The nurseries are under the inspection of the London County Council, and some probationers receive instruction in "first aid" and "home nursing" at the Council schools during winter months. Some experience can also be had in laundry, house, and kitchen, if desired, as opportunity offers. This would seem a good preparation for girls at home or abroad, who are unable to get the experience in their own homes.

"A Constant Reader" writes: In your Note headed "An Impracticable Proposal" you mention the fact that at several institutions "girls can be received for a few months and be trained exclusively in nursing children, both well and ill." I should like to draw the attention of your correspondent to one of those institutions in particular—namely, the one established here in Liverpool under the auspices of the "Liverpool Ladies' Sanitary Association." This provides the hospital training, both theoretical and practical, which your correspondent rightly considers is so essential. The training is intended, naturally, for ladies wishing to be children's nurses, but the Association is quite willing to admit, to the whole or part of it, any ladies who desire to benefit by it without adopting it as a career. In reading your very interesting accounts of "The Waltham System" in two recent issues it struck me that the training here for children's nurses approaches to a great extent that preparatory course upon which your journal frequently lays stress. In fact, some of our children's nurses who have ultimately found their vocation in hospital life have admitted that this preliminary training had helped them wonderfully. The Secretary, L.L.S.A., 8 Sandon Terrace, Liverpool, will be glad to send syllabuses and particulars of training to anyone interested in the work."

### UNEMPLOYED WOMEN.

The Secretary of the Central Bureau for the Employment of Women writes : As there seems no doubt that a large number of well-educated women, as well as women of the industrial class, are at present in need of employment, may I ask you to make known, through the medium of your valuable paper, that at the Central Bureau for the Employment of Women, 9 Southampton Street, High Holborn, information or advice on any subject connected with employment can be had at any time for the small sum of 1s.? We have for seven years carefully weighed and classified all the information we could collect on the professions of women, and we try, whether verbally or by letter, to place it as fully and usefully as possible at the disposal of inquirers.

### THE USE VERSUS THE ABUSE OF WALLETS.

"M. C. H." writes : As a sister of some years' experience, may I venture to suggest that "Anti-Wallet's" sweeping condemnation of those handy receptacles for instruments, and her contention that they are an "impossibility" to a "modern well-trained nurse," need confirmation from other "well-trained nurses"? I believe that a majority will agree with me that a well-kept, well-filled wallet, either on one's person or close at hand, is a boon both to the happy possessor of it and to the visiting surgeon, who haply may need a probe, scissors, or forceps, on his round, and without a rush round to find and collect them, sees them withdrawn from the despised wallet at a moment's notice, and placed in the steriliser, soon to be, by the cleansing power of boiling water, as free from germs through dust or other septic matter as if they were brought direct from the immaculate air-tight glass case, the pride of a theatre sister's heart, but a treasure not always attainable, or, may I add, necessary in all wards of an institution? To be brief, as instruments for surgical purposes must undergo the process of sterilisation, let us not cavil at the long-valued and much-tried wallet, but be thankful that it has largely taken the place of the chatelaine, the bugbear of a patient desiring rest, and a source of irritation to the visitor, who could not but contrast the noisy dangling chains unfavourably with the quietude and noiseless movement, the *sine qua non* of a thoughtful nurse.

### THE LONG VEIL.

"One who is Contented" writes : It was with great interest and amusement that I read the letter about "the long veil" in your columns. I think that the majority of nurses will agree that none but hospital nurses should be permitted to wear the long veil. Moreover, it is becoming, as the writer of the letter in question says, but much depends upon the wearer. I myself wear a veil at the present time, because at the hospital where I had a year's training the veil was a part of the outdoor uniform, and was bought at the expense of each individual. Taking into consideration my hope of entering some other hospital, I feel perfectly justified in wearing it, and trust in time to come that my long veil—well earned—will adorn the head of a good and capable nurse. It would certainly not be given up without a pang. Your correspondent seems to have met with a dreadful catastrophe with the long veil. As yet I have only once had cause to think it a nuisance. One very windy day a few months ago whilst walking down the street, and just about to turn the corner, I suddenly felt a tug at my bonnet. On looking round I found myself a captive, the veil having caught on a lady's hat-pin and entwined itself in the trimming. Whilst we were trying to free ourselves from the entanglement, one of the opposite sex came to the rescue, and quickly set us at liberty. May I conclude with a suggestion? Do you not think a yard and a half too long? One yard, or a little less, in length looks very neat, is less expensive, and certainly does not bring the wearer into such difficulties.

### THE CENTRAL MIDWIVES BOARD AND ASTON UNION INFIRMARY.

"Sister" writes : I am much surprised to hear that Aston Infirmary is not considered suitable as a training school for midwives by the Central Midwives Board, and more than surprised to know the reason. Are not the Board somewhat likely to defeat their own end? I would suggest that Aston Infirmary and other infirmaries of equal size and importance are most suitable for training the class of midwives which the Board most desire. Considering that out of 430 cases there has not, during the last six years, been a single death or case of septicæmia at Aston Infirmary, one is much tempted to question the wisdom of the decision of the Board with regard to the reason which they give—*i.e.*, "that the structure and general condition of the infirmary" are considered unsatisfactory. This is the more to be deplored because the Guardians were willing to make any alterations the inspector of the Central Midwives Board deemed advisable or necessary. Having regard to the primary object of the Board, it seems a pity to stem the source of such appropriate training as many of our large infirmaries offer, without a more serious reason than the one above quoted, which could and would have been surmounted. One cannot help wondering what will happen to some training schools (whose nurses take the greater number of their cases in the slums) if the question of structure and general condition is to detract from a nurse's training. I am forcibly reminded of the proverb dealing with the gnat and camel. It is scarcely consternation which reigns at Aston Infirmary, because the general training given (even without the excellent monthly training and certificate for same, in addition to the three years' certificate) prevents the nurses feeling anything but a natural indignation at the unfair treatment they have received at the hands of the Central Midwives Board. There are women now holding important posts in the nursing world, especially where midwifery is concerned, who were trained at Aston Infirmary, and I sincerely hope that the Board may, early in the future, see their way clear to altering a much-to-be-deplored decision.

### Presentations.

Bath Union Infirmary.—Nurse Cash, deputy-superintendent nurse at Bath Union Infirmary, was recently presented with a handsome clock from the Hospital Committee of the Bath Guardians, a silver teapot from the medical officer and nursing staff; and a pair of bronze figures from the master, matron, and officials of the workhouse, on the occasion of her marriage. She also received various gifts from old fellow-nurses, patients, and patients' friends. Nurse Cash has been at the Bath Union Infirmary for ten years, and her departure is generally regretted.

Morley Convalescent Home, St. Margaret-at-Cliff, Dover.—Miss Capes, the matron of the Morley Convalescent Home at St. Margaret-at-Cliff, who has been appointed matron at Shirley Schools, near Croydon, has been presented by the staff and some of the past and present patients with a carriage clock, and by numerous friends in the village with a silver basket. The presentation was made by the vicar of the parish.

Belvidere Hospital, Glasgow.—An interesting presentation took place at Belvidere Hospital, Glasgow, on Friday last, when Miss Mary Munro, who has been over thirty years matron of the small-pox hospital and is retiring on a pension, was presented with a purse of gold from the medical staff, past and present. Dr. A. K. Chalmers, medical officer of health, in a few excellently chosen words made the presentation. After the ceremony the visitors were entertained to tea and cake by the present matron, Miss E. Blaikie, and the proceedings closed with the annual dance which is given to the nursing staff.

### Notes and Queries.

**REGULATIONS.**

**The Editor is always willing to answer in this column, without any fee, all reasonable questions, as soon as possible.**

**But the following rules must be carefully observed.**

1. **Every communication must be accompanied by the name and address of the writer.**
2. **The question must always bear upon nursing, directly or indirectly.**

**If an answer is required by letter a fee of half-a-crown must be enclosed with the note containing the inquiry.**

*Canada.*

(51) Can you inform me of any journal similar to The Hospital in Canada advertising hospital vacancies, etc.?—*H. M. H.*

The Hospital circulates in Canada, or you might write to *The Canadian Nurse*, 133 East Bloor Street, Toronto, Canada.

*Supplementary Training.*

(52) Would you kindly tell me of any hospital where a supplementary training is given and a three years' certificate granted at the end of two? Will it hold as good as one obtained in one institution? I have had fifteen months' general and three years' fever training.—*E. M. A.*

Could you not return to the hospital where you had your fifteen months' training and complete your three years there? If not, advertise your requirements, but a certificate for an interrupted training or in different hospitals is never as good as a continuous three years in one institution.

*Midwives.*

(53) (1) Do you consider midwifery a remunerative profession, (2) and is the supply of midwives in excess of the demand? If the former, (3) I shall be glad if you will advise me which is the best place at which to be trained.—*F. F.*

(1) It depends upon the practice. (2) At present the supply is not in excess of the demand. (3) You can be trained at any of the large lying-in hospitals or by the Royal Maternity Charity, 10 Delamere Terrace, Paddington, W.

*Convalescent Homes.*

(54) Can you inform me whether there are any convalescent homes for nurses who are knocked up with hospital nursing, and therefore, as they are earning no money, cannot afford to pay for a change themselves?—*H. K. W.*

Write to the Matron of Mrs. Henry's Home, Parkwood, Henley-on-Thames, where tired nurses are received as guests.

*Training.*

(55) Will you kindly let me know whether training at —— Infirmary would be as good as that at —— Hospital?—*Teacher.*

Either is excellent.

*India.*

(56) To whom should I apply for particulars regarding the Lady Ampthill Nursing Institute? I think the nurses are sent out to India, and I am desirous to hear about it as soon as possible.—*Sister.*

Write to Lady Ampthill, Government House, Madras.

*Buenos Ayres.*

(57) I should like particulars about nursing in Buenos Ayres. I am a fully-trained nurse (three years), and am anxious to go out there if I could get an opening. Is it difficult to get into a nursing home or hospital there, and is the pay better than at home?—*E. A. S.*

The matron of the British Hospital, 74 Calle Perdeiel, Buenos Ayres, might give you particulars if you write to her.

**Handbooks for Nurses.**

| | Post Free. |
|---|---|
| "How to Become a Nurse: How and Where to Train." | 2s. 4d. |
| "Nursing: its Theory and Practice." (Lewis.) ... ... | 3s. 6d. |
| "Nurses' Pronouncing Dictionary of Medical Terms." ... | 2s. 0d. |
| "Complete Handbook of Midwifery." (Watson.) ... | 6s. 4d. |
| "Preparation for Operation in Private Houses." ... | 0s. 6d. |

**Of all booksellers or of the Scientific Press, Limited, 28 & 29 Southampton Street, Strand, London, W.C.**

### For Reading to the Sick.

### AT THY FEET.

The source and centre of our joy
Is Christ and Christ alone;
Yet, drawn to Him, our spirits touch,
In worship at His throne.
The broken lines of life converge
At Jesus' sacred feet;
'Tis here His loved ones, sundered far,
In glad communion meet.
Himself all loyal hearts confess
Incomparably dear;
Yet nearer to each other press,
Because to Him so near.

*Lucy Bennett.*

"Christ in us," the hope of glory. Christ in us! What a mistake Christians make when they think of God—of Christ the Son of God—as far off. He is our life; He is within us. This is it which makes it so plain how Christ's example is of use to us. People often argue that if Christ was sinless, His example can be of no use to us. Perhaps not, if He were only example. Perhaps His life would strike us, so pure is it, with nothing else than despair. It would be too high for us to follow. But in fact His example, and His atoning sacrifice also, are only parts of His work. He who acts for us, as our representative, also acts in us, as our new life. That very Jesus whose life we read of in the Gospels—that very Jesus who conquered Satan so that he fled away to hide his head in hell—is both before our eyes as our example, and also in us, by His Spirit, as our new life. It is Christ in us Who forms us by His Spirit inwardly upon the model of the pattern which He showed us outwardly.

How is this to be? By faith, you say. Yes! "Thy faith shall make thee whole." But what is faith? It is as it were the open hand, or open mouth, of the human soul. It welcomes God's promise; it expects His gift. What is the gift which it expects and welcomes? It is the Holy Spirit, the Lord and Giver of life. When Jesus was on earth healing men's bodies, it was their faith which made them whole. But how? Because their faith set free to work upon them the healing "virtue" or power which went out of Christ. That healing virtue or power was the virtue and power of the Holy Ghost in His sacred humanity. And still Jesus makes us whole, in soul and spirit first of all, by bestowing upon us out of His sacred manhood the gift of the Holy Spirit.

But the end of all God's dealings with us is that we should be made at last actually righteous; that we should be rid, not merely of the guilt of sin, but of its power. Everything in religion is indeed a means of this one end—that we may be brought back into actual living union with God, and this, of course, means actual likeness to God. . . . And however weak we are, we have Christ in us, by His Spirit, ready to strengthen us, and make us like Him. Never let us despair then. Let us hold on to Jesus, our example, our Sacrifice, our New Life. There is no failure except in ceasing to try; we can never be lost except by deliberately abandoning God.

*Bishop Gore.*

## Figures and Tables

**Figure f1:**